# Supplementary material for: The lived experience of people affected by cancer: A global cross-sectional survey protocol
Source: PLoS One. 2024 Feb 23;19(2):e0294492. doi: 10.1371/journal.pone.0294492 (PMC10889872; doi:10.1371/journal.pone.0294492)
Supplement: S1 Table — Complete list of items, measures and response options in survey questionnaire. (DOCX) [file pone.0294492.s001.docx]

| **Aim** | **Item** | **Number of items** | **Measure and response option** | **All People affected by cancer** | **Survivors** | **Family members of people affected by cancer** | **Bereaved family members** |
| --- | --- | --- | --- | --- | --- | --- | --- |
| **Introductory questions** | - Items asking If a cancer diagnosis is present in self or family member, and whether the person is receiving treatment. - Items ensuring age above 18 years old | 8 | Forced choice options. Items purposely developed. | **X** | **X** | **X** | **X** |
| **Demographics** | - Age - Gender - Country - First language - Highest level of education - Marital status - Pregnancies and children - Ethnicity - Current employment status - Members of household - Indigenous status - Health insurance status | 18 | Mixture of forced choice options with options to add free text (e.g., ‘other’), depending on the question. Items either purposely developed or based on Childhood Cancer Survivor Study or the FOCUS study. | **X** | **X** | **About self** | **About self** |
| **Clinical information about person with cancer** | - Age at diagnosis - Remission status - List of diagnoses and most recent diagnosis - Cancer type (first and any subsequent diagnoses) - Cancer treatment status and treatments received (e.g., chemotherapy, radiation, surgery, immunotherapy, targeted therapies, transplant) - Cancer predisposition syndrome and cancer genetics | 20 | Mixture of forced choice options with options to add free text (e.g., ‘other’), depending on the question. Items based on the FOCUS study. | **X** | **X** | **About their family member (excluding type of treatments received)** | **About their family member (excluding types of treatment received)** |
| **Survivorship care experiences** | - Information received on survivorship care - Health professionals seen for medical issues - Reasons for not receiving survivorship care - Cancer treatment summary / survivorship care plan received - Barriers to accessing specialist care - Hospital admissions in last 2 years for physical health (1 item) | 10 | Items based on the FOCUS study | **X (information and hospital admission)** | **X (all)** | **-** | **-** |
| **Fertility experiences of person with cancer** | - Fertility preservation received, if any - Satisfaction with fertility information received - Fertility treatments used, if any - Questions on menopause | 11 | Mixture of forced choice options with options to add free text (e.g., ‘other’), depending on the question. Items purposely developed. | **X** |  | **-** | **-** |
| **Perceived impact of cancer** | - Items assessing the impact of cancer and cancer treatment on multiple areas of life, including 1) education plans (2) work life or career (3) ability to date people (4) desire to have children (5) ability to have children (6) relationship with your spouse/partner (7) sex life (8) relationship with children (9) relationship with other family members and friends (10) participation in social activities (11) financial situation (12) diet (13) exercise activities (14) smoking of tobacco (15) alcohol consumption (16) ability to get or retain health, life or disability insurance (17) ability to enjoy life | 24 | Items based on the FOCUS study | **X** | **X** | **-** | **-** |
| **Impact on marriage/ relationship (if applicable)** | - Impact on marriage/ relationship | 6 | Purposely developed | **X** | **X** | **X** | **X** |
| **Education/work problems** | - Ability to work - Ability to study. - Educational support services received - Access to employment, insurance, loans | 6 | Adapted from Long Term Follow Up study questionnaire and purposely developed | **-** | **X** | **Self** | **Self** |
| **Caregiver burden** | - Family caregiving burden - Worry about family member (only for families of current patients or survivors)- purposely developed | 27 | Family Appraisal of Caregiving Questionnaire for Palliative Care (FACQ-PC) by Cooper et al. 2006, and an additional item purposely developed |  |  | **Self** | **Self** |
| **Prolonged Grief** | - Family member’s experiences with symptoms of prolonged grief after the loss of a loved one | 11 | Prolonged Grief Scale by Prigerson et al. 2021 | **-** |  | **-** | **Self** |
| **Impact of cancer on siblings** | - Sibling’s psychosocial and practical needs related to the impact of their siblings’ cancer diagnosis and survivorship - Other impacts on siblings | 24 | Sibling Cancer Needs Inventory (Patterson et al. 2014) and purpose-designed questions (12 items) | **-** |  | **Siblings only** | **-** |
| **Health-related quality of life** | - Physical health, mental health, social health, pain, fatigue and quality of life. | 10 | PROMIS Global | **X** |  | **Self** | **Self** |
| **Health behaviours** | - Alcohol, medication, drugs and exercise behaviours | 4 | Purposely developed | **X** | **X** | **Self** | **Self** |
| **Emotional problems** | - Types of emotional problems and timing | 3 | Purposely developed | **X** | **X** | **Self** | **Self** |
| **Mental health** | - Mental health related hospital admissions in last 2 years. | 1 | Purposely developed | **X** | **X** | **Self** | **Self** |
|  | - Visits to mental health professionals | 2 | Items based on the FOCUS study | **X** | **X** | **Self** | **Self** |
|  | - Anxiety | 8 | PROMIS Anxiety short form | **X** | **X** | **Self** | **Self** |
|  | - Depression | 8 | PROMIS Depression short form | **X** | **X** | **Self** | **Self** |
| **Financial well-being** | - Financial difficulties (e.g., can’t attend care due to cost, financial impact of child loss) - Financial toxicity | 6 | Purposely developed | **X** | **X** | **Self** | **Self** |
|  | - COST measure items assessing worry about future financial situation, satisfaction with present financial situation, cancer hurting financially | 3 | COST measure developed by De Souza et al 2017 | **-** | **X** | **Self** | **Self** |
|  | - COST measure items assessing: no choice about money spent on medical care, frustration about not being able to work, satisfaction with current financial situation, ability to meet monthly expenses, feeling financially stressed, concern about keeping job, feeling in control of financial situation | 7 | COST Measure, developed by De Souza et al 2017 | **X** |  | **Self** | **Self** |
|  | - Source of income during and after treatment | 2 | Purposely developed | **X** | **X** | **Self** | **Self** |
| **Employment supports** | - Questions on: support from employers during and after cancer treatment, access to paid leave, access to financial support for housing/transportation/food, other practical supports | 5 | Purposely developed | **X** | **X** | **Self** | **Self** |
| **Support service use** | - What types of health professionals have been spoken to regarding support needs/mental health concerns - Referrals for support needs/mental health concerns - Barriers to accessing support - Preferences for support | 13 (6 items) | Based on a National Cancer Institute Survey | **X** | **X** | **Self** | **Self** |
| **End of life care experience** | - Timing and age of death - Cause of death (cancer vs treatment) - Professionals seen at end of life - Access to palliative care and advance care planning, and timing of those - Care consistent with wishes - Spiritual wishes - Information received on end of life - Amount of support received - Open-ended question for parents who have lost a child. | 18 | Purposely developed | **-** | **-** | **-** | **Self and family member affected by cancer** |
| **Post-traumatic growth** | - Open-ended questions about other impacts of their/their family member’s cancer experience | 2 | Purposely developed | **X** |  | **Self** | **Self** |
| **Missed topics** | - Open ended - questions that were not asked in this survey | 1 | Purposely developed | **X** |  | **Self** | **Self** |

**S1 table. Items and measures in survey questionnaire.**
