# Supplementary material for: The lived experience of people affected by cancer: A global cross-sectional survey protocol
Source: PLoS One. 2024 Feb 23;19(2):e0294492. doi: 10.1371/journal.pone.0294492 (PMC10889872; doi:10.1371/journal.pone.0294492)
Supplement: S2 Table — Stakeholders per geographical region and focus population. (DOCX) [file pone.0294492.s002.docx]

| **Institution/ Organisation** | **Target region** | **Focus population** |
| --- | --- | --- |
| National Cancer Institute (NCI) | United States of America | All cancers |
| Union International contre le Cancer (UICC) | All WHO regions | Adult cancers, with a focus on women’s cancer  Childhood cancer |
| Childhood Cancer International (CCI) | All WHO regions | Childhood cancer |
| International Psycho-oncology Society (IPOS) | All WHO regions | All cancers |
| American Childhood Cancer Organization (ACCO) | North America | Childhood cancer |
| International Society of Paediatric Oncology (SIOP) | All regions | Childhood cancer |
| Teal Sisters Africa | Africa | Women’s cancer |
| New Sunshine Charity Foundation | China | All cancers |
| CanKids | India | Childhood cancer |
| Faros de Vida | Central and South America | All cancers |
| International Society of Geriatric Oncology (SIOG) | All WHO regions | Adult cancers |
| WHO Regional Offices | ALL WHO regions | All cancers |

**S2 table. Stakeholders supporting study dissemination.** Stakeholders per geographical region and focus population.
