# Supplementary material for: The lived experience of people affected by cancer: A global cross-sectional survey protocol
Source: PLoS One. 2024 Feb 23;19(2):e0294492. doi: 10.1371/journal.pone.0294492 (PMC10889872; doi:10.1371/journal.pone.0294492)
Supplement: Attachment — Submitted filename: Review PLOS One.docx [file pone.0294492.s003.docx]

Review PLOS One

**The lived experience of people affected by cancer: A global cross-sectional survey**

**protocol**

The authors are planning a survey including cancer patients and their relatives distributed a global scale. Their main aim is to better understand psychosocial and financial aspects of the lived cancer-related experience journey. The sample will include persons living with cancer or in longterm survivorship and family members of a person while they are alive as well as bereaved family members.

The authors are highlighting a lack of available global data on the topic in general and in LMIC especially. The topic clearly is of great importance and the study will create helpful and interesting information for institutions and individuals working with cancer patients or in health system planning.

The background section starting as line 62 gives a comprehensive overview on the psychosocial and financial issues cancer patients and relatives are facing according to the literature. While the structure is clear and good to follow, it is quite lengthy by writing style and the information could be conveyed in a more compact manner.

The highlighting of the lack of data from LMIC both in abstract (lines 40; 50) and main text (e.g., lines 226; 240) and the studies aim to add new information to the literature are positive. The inclusion of high-income countries and LMIC will help determining differences between countries and regions. Nevertheless, the representation of the LMICs seems it will be lower in the study. Since the authors stated the special attention needed in LMIC, further inclusion of such settings in the ongoing planning process seems advisable if resources exist. It is advisable to follow the stated further inclusion of stakeholders from underrepresented regions (lines 280 - 286). While there is an international collaboration working on the project, affiliate members from regions Asia and South- and Central America seem missing, especially since the questionnaire will be available in Spanish and Chinese.

*Lines 281 to 284 (invite additional members as needed, including, for example, underrepresented groups of people with lived experience (e.g., fathers, culturally diverse people, First Nations people):* It is a challenging but important endeavor. I suggest making and publishing a plan how to deal with this situation in a structured manner. This will also increase transparency.

Moreover, the recruitment plan (lines 353 ff.) seems to involve effective strategies with WHO support and via other institution. It might be helpful to publicize a strategy on how to recruit participants from the worlds region good receive adequate representation of the regions. Otherwise be more specific about the target regions.

It would be interesting to learn more about the methods of translating the questions into the different languages.

Data collection seems to be anonymously if the participant does not agree for a follow-up. How is personal data handled for those cases?

Learning more from the participants in qualitative follow-ups seems very informative. It would be interesting to learn in more detail about the methods planed for qualitative follow up interviews especially since the number of participants could become very high.

The planned sample size is clearly stated (n=2000) and calculates for a subgroup of 1000 participants indicating a binary subgroup analysis (lines 409 ff.). However, the planned target groups are 4 (lines 324 ff.) and multiple further subgroup analyses are mentioned (disease and age groups, countries: lines 398 ff.; time since diagnosis line 426). It would be interesting to learn more about the calculations giving these circumstances as well as the planned definitions of the disease/age subgroups (there are multiple cancer entities, that largely differ in the way they affect patients’ health and with it their individual needs).

This would support the mentioned development of the more detailed analysis plan (line 428-430).

Concerning the distribution, those seem reasonable. It might be fair to participants and seemingly manageable to offer the option of sending the planned easy to understand summary of the studies’ findings at the end of the online questionnaire.

Overall, the scientific community can look forward to see results from this upcoming study.
